# Separation and not residency permit restores function in resignation syndrome: a retrospective cohort study

**DOI:** 10.1007/s00787-021-01833-3

**Published:** 2021-07-05

**Authors:** Karl Sallin, Kathinka Evers, Håkan Jarbin, Lars Joelsson, Predrag Petrovic

**Affiliations:** 1 Centre for Research Ethics and Bioethics (CRB), Uppsala University, P.O. Box 564, 751 22 Uppsala, Sweden; 2 K8, Department of Clinical Neuroscience, Karolinska Institutet, 171 77 Stockholm, Sweden; 3 Q2:07, Department of Paediatric Neurology, Astrid Lindgren Children’s Hospital, Karolinska University Hospital, 171 76 Stockholm, Sweden; 4 Faculty of Medicine, Department of Clinical Sciences Lund, Child and Adolescent Psychiatry, Lund University, Box 117, 221 00 Lund, Sweden; 5 Child and Adolescent Psychiatric Clinic, Uddevalla Hospital, Fjällvägen 9, 451 53 Uddevalla, Sweden

**Keywords:** Apathy, Malingering, Refugees, Family separation

## Abstract

Despite poor treatment results, a family-oriented approach and the securing of residency have been deemed essential to recovery from resignation syndrome (RS). In a retrospective cohort study, we evaluated an alternative method involving environmental therapy, with patients separated from their parents, while actively abstaining from involving the asylum process in treatment. We examined medical records, social services acts, and residential care home acts from 13 individuals treated at Solsidan residential care home between 2005 and 2020. Severity and outcome were assessed with Clinical Global Impression, Severity and Improvement subscales. Thirteen participants were included and out of these nine (69%) recovered, i.e. they very much or much improved. Out of the eight that were separated, all recovered, also, one non-separated recovered. The difference in outcome between subjects separated and not was significant (*p* = 0.007). Moreover, out of the five which received a residency permit during treatment, one recovered whereas four did not. The difference in outcome between subjects granted residency and not was significant (*p* = 0.007). The data revealed three (23%) cases of simulation where parents were suspected to have instigated symptoms. Our evaluation suggests that separation from parents and abstaining from invoking residency permit could be essential components when treating RS. Relying on a family-oriented approach, and residency could even be detrimental to recovery. The examined intervention was successful also in cases of probable malingering by proxy.

**Supplementary Information:**

The online version contains supplementary material available at 10.1007/s00787-021-01833-3.

## Introduction

The first noted case of resignation syndrome (RS) appeared 1998 in northern Sweden. While awaiting a residency permit, a boy of Chechnyan descent deteriorated and was left bed-ridden, unresponsive and in need of feeding to sustain. After the family was granted residency the boy recovered. More cases ensued in the same region [1], and later throughout the country. A peak prevalence of 424 cases was observed January 1st, 2003 to April 30th, 2005 [2]. In 2014, the Swedish National Board of Health and Welfare introduced the label “Uppgivenhetssyndrom” (resignation syndrome) and a national ICD-10 code, F32.3.A, categorizing the condition among the depressive disorders. A recent official estimate numbered 414 individuals (2014–2019) [3]. Among asylum-seeking children and adolescents under psychiatric care in Sweden during 2014, 5.1% suffered from RS [4]. Diagnostic criteria remain undetermined, however, see supplementary material for proposals. In 2018, suspected cases in a migrant population were reported from the Island of Nauru. These cases have also been labelled pervasive refusal syndrome (PRS) [5], a condition resembling RS but involving active refusal. Neither are recognised by international classification systems. PRS occurs worldwide, however, mostly as isolated cases [6]. The RS endemic in Sweden, involving more than a thousand patients and now in its third decade, lacks international comparison.

Onset usually follows a negative event, such as a rejected asylum application [7, 8]. Failure to ingest prompts hospitalisation, medical work-up and eventually tube-feeding. The ensuing out-patient intervention further involves prophylactic physiotheraphy and therapeutic sensory stimulation including the upkeeping of a daily routine. The psychiatric pharmacopoeia, although unevaluated systematically, has not appeared beneficial. The family is responsible for administering treatment and is moreover considered instrumental in its capacity to convey hope and reassurance [9].

Recovery is often slow, sometimes tube feeding endures for several years [8, 10]. In-patient samples exhibit shorter tube-feeding periods (*n* = 6, median 10 weeks [4–24] [11], *n* = 5, mean 27 weeks (10–60) [12]) than out-patient samples (*n* = 22, mean 46 weeks (5–107) [10], *n* = 12, median 40 weeks [17–56] [11]). Return of function has been reported in a sequence of gross motor skills, fine motor skills, eye contact and last, communication [10]. Long-term follow-ups are few but indicate a return to normal function [12]. A residency permit has been asserted essential for recovery [9].

Many hypotheses regarding the nature and causes of RS have been put forward. The conception of stress-induced functional loss resulting from previous trauma coupled with the threat of expulsion predominates [13, 14]. However, the endemic nature of RS, essentially a Swedish phenomenon and selectively striking immigrant populations from certain parts of the world, remains unexplained on this account. Hence, in line with a model of functional symptoms [15], the notion of a culture-bound disorder was invoked and suggested to be the upshot of expectations coded in the brain as priors [16], an approach taken also to e.g. autism [17], psychosis [18] and depression [19]. Some accounts of RS include malingering, which recently was unveiled in two cases [20].

The patient group marginalized and notoriously difficult to enroll in studies, RS has not received sufficient scientific attention. There are no intervention studies that target treatment methods, something recently noted by the Swedish Agency for Health Technology Assessment [21]. Instead, methods to sustain somatic health and reverse loss of function are supported by clinical practice and descriptive studies. Notably, a national guideline, asserting residency and parental involvement to be essential for recovery, was issued in 2013 [9].

The residential care home Solsidan, treating patients either on voluntary admission or after a court ruling relieving parents of legal custody, and hence of the right to refuse treatment on behalf of their child, utilises a different methodology. Three principles guide the approach: residential therapy, separation, and active abstaining from involving the securing of a residency permit. The latter two are unique to Solsidan–and contrary to the national guideline [9]–while stimulation is practiced elsewhere, however, unlikely equally intensely. Interestingly, separation, although controversial, has historically been proposed effective in treating paediatric hysteria [22, 23] and is contemporarily recommended to counter medically unexplained symptoms in children [24].

The Solsidan method (see Box 1) and an anectdotal narrative of its effects have already been communicated [25]. However, lack of systematic rigor, scarcity of facts and possibility of bias, as the informant has ties to the residential care home, warrants a systematic evaluation.

Box 1: Solsidan: treatment, staffing and general information [25] Solsidan opened in 1934, consists of two wards and admits twelve patients with or without accompanying parents. It is currently operated by Gryning Vård, a publicly owned and operated company. Treatment for RS started 2004. Initially, the whole family was admitted and there was no progress or even deterioration. After a successful treatment attempt involving separation this procedure became standard.

The staff includes social workers, special educators, preschool teachers and social psychologists specially trained in residential theraphy and developmental psychology. On a regular basis, the team is counselled by a psychologist specialised in residential theraphy. The staff is regularly involved in internal meetings with patient-related discussions but also involving giving and receiving support from one another.

The method is characterised as “environmental theraphy” and is devised so as to be contact enabling, containing and stimulating. The staff forms credible and stable relations and structures which specifies and habours the patient’s problems thereby catering for security and predictability. While the staff serve as role models, the stuctures help make problems explicit enabling them to be labelled and countered. The environmental therapy is complemented by observations, patient consultations, family consultations, couple consultations, family therapy, network cooperation work and more.

Medical consultation is provided by a general practitioner also catering for dietician, physiotherapist and occupational therapist. A paediatrician is responsible for tube feeding.

A start up meeting is held between the child, parents and designated members of staff serving as primary contacts. Information is given regarding the treatment plan, daily routine, and family participation. Professional interpreters are present.

If the whole family is admitted (often the case when treatment is initiated), a weekly schedule, specifying what is expected and when is communicated. The daily routine typically includes meals, family activities, “standing and walking”-training, walks, games, bedtime story, TV, personal hygiene, housekeeping, school, and excursions. Weekly meetings are held with the patient. At first, these are conducted as monologues.

At the outset, it is made clear to the family that the asylum process will not be discussed with the patient. Also, the parents are urged to refrain from doing so.

On a weekly basis, meetings with the parents are conducted. These provide opportunities for parents to receive support, discuss their own problems, the asylum process, and the child’s condition.

Throughout separation, which usually lasts 2–3 months, regular reports, including photographs and films, are conveyed to the parents in order for them to remain involved in the process.

Fundamental to treatment is the conviction that the child’s physical health is intact, and that the behavioural change results from an untenable situation. The child is “lured back to life” through stimulation, challenges and the elicitation of frustration. Stimulation involves all senses; “breeze through one’s hair”, “the sound of other children swimming”, “the smell of freshly baked bread”. Challenges and frustration involve not arranging a comfortable seating position and pretending to not understand subtle signals to evoke a stronger reaction. The patient participates in all activities. If a function is lacking, the staff steps in to enable participation. This includes everything from sitting at the dinner table, preparing food, participating in board games etcetera. At all times the patient is addressed as if fully awake and involved in whatever goes on.

Once the patient starts to subtly make the staff conscious of him or her being aware, challenges are posed, tricks are played, and frustration is elicited. This stage in treatment is delicate but when undertaken at a proper pace, partial restoration of function ensues step by step.

When physical function has been restored, separation is gradually broken. This often results in a temporary relapse in one or several functions. Depending on reaction the rate at which contact is normalised is adjusted. At this point, parents are again urged to select topics for discussion with care so as to not evoke distress. Eventually, information regarding the asylum process is given by a member of staff.

During treatment the staff serves as an “emotional crutch”—“someone who knows life can be worthwhile, and challenges and stimulates the child to believe the same”. The staff, unlike the parents, “is capable of being emotionally available and at the same time anchored in life as it proceeds that very moment”.

### Aims of the study

To retrospectively evaluate the treatment method involving separation, residential therapy and abstaining from securing residency, as practised at the Solsidan residential care home, in patients with resignation syndrome. Recognising the anectdotal evidence [25], our hypothesis was that separation, residential therapy, and abstaining from involving residency would enable recovery.

## Materials and method

### Participants

Out of 15 individuals treated at Solsidan (Skara, Sweden) between 2005 and 2020, 13 (87%), nine male and four female, aged 8–15 years, were included. Two subjects were excluded as no records could be retrieved. The participants were referred from six Swedish municipalities, and either committed to, or offered, social preventive care.

### Methods

Items to be analysed (Table 1) were determined prior to data collection. Subjects were identified by name, date of birth, personal identification number, and/or temporary identification number. Data consisted of medical records, social service acts, and acts from Solsidan.

**Table 1:** Items predetermined for analysis

Origin
Minority status
Ethnicity
Religion
Culture
mother tongue
Age
Sex
Previous somatic illness
Previous psychiatric illness
Family constitution
Order among siblings
Education level
Parental function
Somatic illness in the family
Psychiatric illness in the family
Trauma in country of origin stated (yes/no)
Triggering factor (yes/no)
Previous episode of RS (yes/no)
*Level of function on admission*
Responsiveness (yes/no)
Tube feeding (yes/no)
Any communication (yes/no)
Verbal communication (yes/no)
ADL assistance (yes/no)
Duration of RS on admission (days)
Diagnosis
Duration of treatment (days)
Admission to oral intake (days)
Admission to unassisted walking (days)
Admission to communication (days)
Admission to verbal communication (days)
Duration of tube feeding in total (days)
Duration of tube feeding from admission (days)
*Treatment*
Environmental therapy (yes/no)
Separation (yes/no)
Abstaining from involving residency (yes/no)
Treatment effect
Effect of terminating separation

Data were extracted by two examiners blinded to each other (KS and LJ) and results compared. Divergences in quantitative data extraction were settled through a joint rereading of relevant material. Divergences in qualitative data extraction, i.e. rating, were settled through deliberation.

Clinical Global Impression-Severity (CGI-S) and Clinical Global Impression-Improvement (CGI-I) scales[26], (Box 2), were used to rate severity on admission and treatment effect, respectively. CGI-I 1 and 2 were coded as improved (recovery), 3 and 4 as not improved (no recovery). Parental function was rated either as severely impaired, indicating parent hospitalised or equivalent, impaired, indicating reduced capacity, or normal.

Box 2. Clinical global impressions-severity and improvement scales Clinical Global Impressions-Severity Scale (CGI-S):

- 0 Not assessed
- 1 Normal, not at all ill
- 2 Borderline mentally ill
- 3 Mildly ill
- 4 Moderately ill
- 5 Markedly ill
- 6 Severely ill
- 7 Among the most extremely ill of subjects

Clinical Global Impressions-Improvement Scale (CGI-I):

- 0 Not assessed
- 1 Very much improved
- 2 Much improved
- 3 Minimally improved
- 4 No change
- 5 Minimally worse
- 6 Much worse
- 7 Very much worse

### Procedures

Contact with a primary health care centre, paediatric units, child and adolescent psychiatric units, municipal social services and Solsidan was made by telephone, email, or mail. Data collection lasted two years and terminated September 2020.

Items predetermined for analysis were successfully elicited with a few exceptions. Ethnicity, religion, culture, and mother tongue were excluded from analyses due to lack of data. Data on minority status and education level were available for most subjects and included in the analyses. Long-term follow-up data were available for a minority of participants, and therefore not subject to analysis.

Differences in CGI-S and CGI-I rating between the examiners occurred twice in a total of 26 assessments and never exceeded one unit.

Data revealed information warranting post hoc analyses. Prior to 2006, separation had not been practised. Moreover, residency had been granted some patients. Consequently, the comparisons between separated and non-separated participants, as well as between participants granted and not granted residency were made possible, and a control design thereby approximated. Respecting our initial hypothesis, the post hoc hypotheses were that separation and not being granted residency both enable recovery. Due to separation, two subjects granted residency were uninformed of the decision and consequently coded as no residency in the analyses.

Further, the treatment group was found to exhibit heterogeneity with regards to the nature of the behavioural change on which the diagnoses were based. Some subjects, the records stated explicitly, feigned RS. Consequently, particular effort was put into extracting information pertaining to the nature of the behavioural change including malingering, and malingering by proxy.

Two patients had not received an RS diagnosis. In recognition of typical and substantial functional loss these were nevertheless included.

### Ethics

Ethical approval was obtained from the regional ethics review board in Stockholm, dnr 2018/717-31/2.

### Statistics

The software RStudio Desktop (RStudio, Boston, USA) was used for statistical analysis including exploration of possible confounding variables. For comparison of categorical variables Fisher’s exact test was used and for numerical variables the non-parametric Mann–Whitney *U* test. Differences with a *p* value < 0.05 were considered statistically significant.

## Results

### Background information

Subjects originated predominately from former Soviet republics, and former Yugoslavia. All were migrants but one, born in Sweden to asylum-seeking parents. Trauma prior to migration was stated in seven cases. Four participants were from minority groups, but data were scarce pertaining to this item. The mean age was 12 (SD 2.0) years. Nine were boys. Nine were the oldest among siblings. None were an only child. Nine were from two-parent families. The studied group contained three pairs of siblings. Previous psychiatric illness (post-traumatic stress disorder, stress reaction, mutism, stuttering) was found in three participants, and previous somatic illness (nephrotic syndrome, abdominal pain) in two. One participant was diagnosed with autism after discharge. School function data were available for seven participants, and five of these were reported to have functioned well in Swedish schools. None of the subjects had received a residency permit when treatment commenced. In three participants, onset was preceded by a suspected trigger–a negative asylum decision in two cases, and an interview at the migration agency in one. None included were previously afflicted with RS. (Tables 2 and 3).

**Table 2:** Background information, Function on admission, Treatment, Outcome. Stratified by Not improved and improved

*n*	Not improved*	Improved**	*p*
	4	9	
*Background information*			
Origin (%)			0.471
Former Soviet Union	2 (50.0)	7 (77.8)	
Former Yugoslavia	2 (50.0)	1 (11.1)	
Turkey	0 (0.0)	1 (11.1)	
Minority status (%)	0 (0.0)	4 (44.4)	0.228
Age at admission (mean (SD))	12.00 (2.16)	11.33 (2.06)	0.606
Male (%)	4 (100.0)	5 (55.6)	0.228
Previous somatic illness (%)	1 (25.0)	1 (11.1)	1.000
Previous psychiatric illness (%)	1 (25.0)	2 (22.2)	1.000
Oldest among siblings (%)	2 (50.0)	7 (77.8)	0.530
Psychiatric illness in the family (%)	4 (100.0)	8 (88.9)	1.000
Somatic illness in the family (%)	2 (50.0)	0 (0.0)	0.077
Trauma mother country stated (%)	2 (50.0)	5 (55.6)	1.000
No Residency permit when admitted (%)	4 (100.0)	9 (100.0)	NA
Triggering factor (%)	0 (0.0)	3 (33.3)	0.497
No previous episode of RS (%)	4 (100.0)	9 (100.0)	NA
Committed to social preventive care (%)	1 (25.0)	4 (44.4)	1.000
Offered social preventive care (%)	3 (75.0)	5 (55.6)	1.000
*Function on admission*			
CGI severity at admission (mean (SD))	6.25 (0·96)	6·33 (0.71)	0.863
Responsiveness on admission (%)	2 (50.0)	1 (11.1)	0.203
Need of ADL assistance on admission (%)	4 (100.0)	9 (100.0)	NA
Oral intake on admission (%)	2 (50.0)	5 (55.6)	1.000
Unassisted walking on admission (%)	2 (50.0)	1 (11.1)	0.203
Verbal communication on admission (%)	1 (25.0)	0 (0.0)	0.308
Communication on admission (%)	2 (50.0)	3 (33.3)	1.000
Tube fed on admission (%)	2 (50.0)	4 (44.4)	1.000
Duration of RS on admission in days (median [range])	50.00 [7.00, 93.00]	98.00 [0.00, 1379.00]	0.353
*Treatment*			
Separation (%)	0 (0.0)	8 (88.9)	0.007
Residency permit during treatment (%)	4 (100.0)	1 (11.1)	0.007
*Outcome*			
CGI improvement at discharge (mean (SD))	3.75 (0.50)	1.11 (0.33)	< 0.001
Duration of treatment admission to discharge in days (median [range])	306.50 [242.00, 371.00]	137.00 [80.00, 620.00]	0.063
Oral intake			
Oral intake at discharge (%)	4 (100.0)	9 (100.0)	NA
Admission to oral intake in days (median [range])	142.50 [137.00, 148.00]	30.50 [2.00, 102.00]	0.064
Unassisted walking			
Unassisted walking at discharge (%)	2 (50.0)	9 (100.0)	0.077
Admission to unassisted walking in days (median [range])	NA [Inf, −Inf]***	34.50 [6.00, 297.00]	NA
Verbal communication			
Verbal communication at discharge (%)	1 (25.0)	9 (100.0)	0.014
Admission to verbal communication in days (median [range])	NA [Inf, −Inf]***	39.00 [11.00, 154.00]	NA
Communication			
Communication at discharge (%)	2 (50.0)	9 (100.0)	0.077
Admission to any communication in days (median [range])	NA [Inf, −Inf]***	23.00 [1.00, 102.00]	NA
Tube feeding			
Tube fed at discharge (%)	0 (0.0)	0 (0.0)	NA
Admission to tube removal in days (median [range])	232.50 [189.00, 276.00]	44.00 [8.00, 102.00]	0.064
Total duration of tube feeding in days (median [range])	281.50 [195.00, 368.00]	230.00 [109.00, 1406.00]	0.643

^*^CGI improvement score 3 and 4

^**^CGI improvement score 1 and 2

^***^No subject attained function during treatment

**Table 3:**
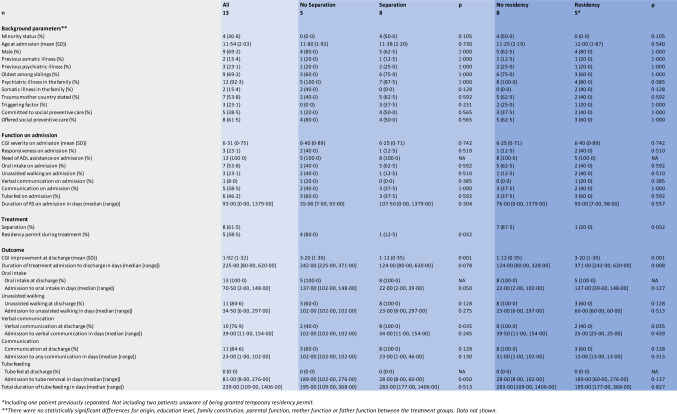
Comparison between treatment groups

In all but one family, reduced parental capacity was noted. In five cases the mothers were severely impaired due to depression or RS, in some cases warranting hospital admission. In seven cases mother function was impaired, in the records a passive or overprotective attitude was typically noted. Out of the nine fathers, one was noted severely impaired, diagnosed with schizophrenia, five impaired due to stress and three exhibited normal capacity. In all but one case, at least one parent had reported or was treated for, psychiatric symptoms. (Tables 2 and 3).

### Function on admission

On admission, six participants were tube fed and one spoon fed. Ten were unresponsive. Twelve failed to communicate verbally, and eight were incapable of any communication. The repertoire of communicative acts was limited in those where function remained. All relied on extensive support in activities of daily living (ADL). Ten were unable to walk unassisted. Six were incontinent. Participants had been suffering from RS for a median of 93 [0-1379] days on admission. Two had not been diagnosed with RS but suffered from prodromal symptoms, and were severely impaired (CGI-S 5 and 6, both in need of ADL assistance, one unresponsive, none communicated verbally, one non-verbally, one walked unassisted, none were tube-fed) (Tables 2 and 3).

Data on medical work-up was incomplete. Findings reported included: contractured ankle, skin marks bordering pressure ulcers, lack of gag reflex, weight and muscle mass loss; preserved bowel and bladder function when assisted to the toilet, ability to shift position while sleeping, at times maintaining eyes half open but nevertheless unresponsive; aggression towards the mother prior to becoming bed ridden and unresponsive; cooperation in feeding and ADL, unresponsive.

Five participants were committed to compulsory social preventive care, and eight were on voluntary admission.

### Treatment

All participants were subject to residential therapy. Eight (62%) were separated, five were not. Five (38%) were granted residency, six were not. Two were granted temporary residency but not informed (see “Methods”; “Procedures”). One (8%) was separated and later, after relapsing when separation had ended, granted residency. One (8%) received only residential therapy. The group separated and the group granted residency were mutually exclusive save for one subject. See Table 4 for an overview of morbidity, treatment, and outcome. See Box 1 for a description of the treatment method.

**Table 4:** CGI score on admission, at discharge and treatment method for each patient (columns)

	Subjects												
CGI severity (0–7), at admission	7	6	6	7	6	6	7	5	7	7	5	6	7
Separation (s), residency permit (r)^†^		s	s	s	s	s	s‡	s‡	s r	r	r	r	r
CGI improvement (0–7), at discharge	1	1	2	1	1	1	1	1	1	4	4	4	3

^†^All received residential therapy

^‡^Received temporary residency permit but remained unnotified during treatment

### Outcome overall

Treatment lasted for a median of 225 [80–620] days (32.1 weeks) (admission to discharge) and, when applicable, included time in and out of separation, time in gradual discharge, and time spent in placement at a family home. In the six tube-fed participants, a median of 81 [8–276] days (11.6 weeks) passed until the tube was removed. In one, the tube was reintroduced after 421 days and later again removed. Duration of tube feeding, including the period prior to admission, was in median 239 [109–1406] days (34.1 weeks). (Table 3).

Nine participants (69%) improved [CGI-I mean 1.11 (SD 0.33)] and four (31%) failed to improve [CGI-I mean 3.75 (SD 0.50)] during treatment (Table 4). Neither function on admission nor background parameters differed significantly between the groups. (Table 2).

### Outcome of separation

The group separated (*n* = 8) and the group not separated (*n* = 5) did not differ significantly in background parameters, or in function on admission. (Table 3).

**Fig. 1:**
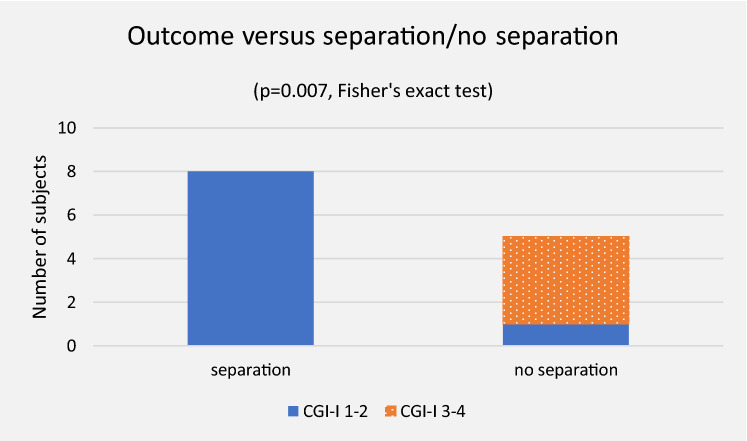
Separation and outcome

Outcome differed in favour of the separated group with a CGI-I mean of 1.12 (SD 0.35) versus 3.20 (SD 1.30) (*p* = 0.001). All separated subjects recovered. Only one subject not separated recovered after what, according to the records, could be interpreted as a panic attack. This participant was not granted residency during treatment. (Tables 3 and 4, Fig. 1).

In the separated group, all functions were regained. In the non-separated group, one out of four regained speech while one spoke on admission, one out of three regained non-verbal communication while two communicated non-verbally on admission, and one out of three regained unassisted walking while two walked unassisted on admission.

There was a strong trend towards a shorter time from admission to oral intake in the separated group with a median of 22.0 [2–39] days, compared to the non-separated with a median of 137.0 [102–148] days (*p* = 0.050). The same held true for time from admission to tube removal; 28.0 [8–60] versus 189.0 [102–276] days in median (*p* = 0.050).

On termination of separation three participants relapsed temporarily, three were unchanged, and one content on being reunited. In one separate case, no data were retrieved indicating the effect of reuniting with the family.

### Outcome of residency permit

The group granted residency (*n* = 5) and the group not granted residency (*n* = 8) did not differ significantly in background parameters, or in function on admission (Table 3).

**Fig. 2:**
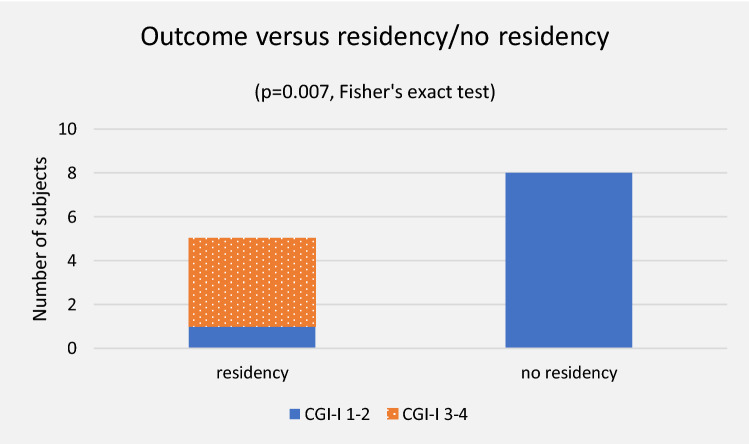
Residency and outcome

Outcome differed in favour of the group not granted residency with a CGI-I mean of 1.12 (SD 0.35) versus 3.20 (SD 1.30) (*p* = 0.001). One subject granted residency recovered. This participant had previously been separated (Tables 3 and 4, Fig. 2).

Duration of treatment was longer in the group granted residency with a median of 371 [242–620] versus 124 [80–328] days (*p* = 0.008).

In the group not granted residency, all functions were regained. In the group granted residency, one out of four regained speech while one spoke on admission, one out of three regained non-verbal communication while two communicated non-verbally on admission, and one out of three regained unassisted walking while two walked unassisted on admission.

### Group heterogeneity

All participants, except for two with severe prodromal symptoms, were diagnosed with RS. However, the data suggested heterogeneity with regards to the nature of the behavioural changes on which the diagnoses were based.

One participant, characterized as immobile and incontinent, was, according to the records, surprised by a member of staff while running across the floor, and when caught in the act, quickly returned to bed. It was documented that the incident was taken as evidence of child abuse, which was reported to the police. The family was later expelled.

In a second case, the records state as a fact that the patient “had acted ill and in resignation”. The informant considered contacting the police.

In a third case, the records state the story given by the parents to be “remarkable”, and “incredible”. They had, the records state, refrained from seeking help despite the severe deterioration they stated their child had suffered, and had claimed unawareness of where to seek help, although they at the time resided next door to the migration agency office. The child was committed to immediate social care.

Based on the assertions made in the records, three (23%) of the participants feigned RS. Two of these were tube-fed on admission. All were separated, one also received residency. All three recovered. The two tube-fed subjects were relieved of their nasogastric tubes after eight days and 60 days, respectively.

In five cases (38%), a clear negative but not apparently deceptive effect from caregivers was indicated in the records. In particular, what could be interpreted as extreme over-protectiveness and in two cases a “symbiotic” relation between mother and child, was noted and claimed to interfere with treatment.

In the remaining five cases (38%) the parental function was reduced in all but one (see “Background information”). The data revealed no proof of an apparent negative effect in these four (31%) cases, save for what was reflected in measures taken by the social services.

## Discussion

We have presented a retrospective evaluation of residential therapy, separation, and abstaining from invoking residency permit in eleven patients with RS, and two with prodromal symptoms, treated at Solsidan residential care home. Counter to the general presumption [9], being granted a residency permit was not related to improvement whereas separation from parents was.

Health issues and suffering among children and adolescents undergoing migration and asylum seeking are substantial and multifaceted yet the scientific literature is scarce [27]. This unfortunate circumstance applies also to RS and to our knowledge, there are no previous studies involving separation in a similar context, nor for that matter in children suffering from medically unexplained symptoms, conversion disorder or functional neurologic symptom disorder.

The examined cohort resembles those previously described [10, 12, 28] in terms of background characteristics and the functional loss, with two exceptions. Mean age, 11.5 compared to 13.6 [12] and 14.4 [10] in other cohorts, differs most likely due to admission restricted to those below 15 years of age. Although from our experience not a rare circumstance, commission to social preventive care has not been noted in previous materials.

All participants were rated severely to extremely ill at admission, while most patients were recovered at discharge. Hence the method seemed to be effective but time consuming, with a median of 7 months. Duration of tube feeding from admission to tube removal was within the range of previous in-patient materials [11, 12], and shorter than in out-patient cohorts [10, 11]. The sequence of recovery starting with non-verbal communication, followed by unassisted walking, later verbal communication and finally oral intake, differed from a previous cohort [10] where the authors proposed the sequence to suggest organic pathophysiology.

To our surprise, the material comprised not only separated patients not granted residency, but also non-separated patients, and patients granted residency during treatment. Enabling the approximation of a controlled design, this urged key post hoc analyses.

Some of the non-separated patients failed to regain non-verbal and verbal communication as well as unassisted walking, whereas recovery was complete in the separated group. Only one patient granted residency recovered, and that patient had previously been separated. Also, there was a strong trend towards shorter duration until oral intake in the separated group and a significantly shorter duration of treatment in the group not granted residency. Thus, consistent with our original hypothesis, these post hoc findings suggest that separation promotes recovery whereas a residency permit obstructs it.

Moreover, a detailed analysis of the subgroups supplies additional support. When comparing the period from admission to the removal of nasogastric tube in the separated group and the group not granted residency, both with a median of four weeks (Table 3), to previous in-patient cohorts, both not separated and having recovered after granted residency, with a median of ten [11] and a mean of 27 weeks [12] of tube feeding, the outcome of the studied method, as devised, i.e. separation and abstaining from residency, surpassed that of the previous in-patient cohorts. Also, the total duration of tube feeding (including the period prior to admission) in these two sub groups was in both median 40 weeks, hence longer than the durations in the previous in-patient materials. This presumably indicates our groups to be at least equally, if not more, severely affected than previous cohorts, further strengthening our hypotheses.

Importantly, however, recognising the overlap between groups (see Table 4), our retrospective evaluation cannot determine whether the outcome resulted from receiving residential treatment while separated from parents, from not receiving a residency permit, or from both.

Several findings suggest that separation may influence positively through inhibition of a harmful family dynamic. All families were subject to interventions from social services to secure the wellbeing and safety of the patient. The parental function was reduced in all but one case. An apparent negative family impact was noted in five cases, and on terminating separation three patients relapsed temporarily. The records suggest that three patients were subject to malingering by proxy. Previously, a harmful family dynamic has been endorsed in several multifactorial models of RS [13, 14, 29] and suggested, through impact on priors in a predictive model of brain function, to alter expectations resulting in RS [16], in parallel to a previous account of functional disorders [15]. Further, overprotectiveness, persisting somatic illness attribution and secondary gain was reported to obstruct recovery from functional somatic symptoms in children [30]. Moreover, familial overinvolvement, along with criticism and hostility, conceptualised as expressed emotion, predicts relapse in a range of psychiatric disorders as well as poor treatment effect [31].

Separating children from their parents is controversial. It needs to be reiterated that in the studied material, decisions to separate were made after a social service appeal in court relieving parents of custody–an intervention motivated by deterioration conceived irreversible by other means. This is the standard legal procedure in Sweden when parental caregiving capacity is flawed or when parents are harming their child in other ways.

As far as we can tell, the separations we have studied were performed with the child’s best interest in mind and should not be confused with other contemporary examples of family separation in migrant populations [32]. In fact, although to our knowledge rarely practiced, separation has previously been suggested potent in severe cases of pediatric hysteria [22, 23] and as a component in multimodal rehabilitation of medically unexplained symptoms in children [24].

Apart from the interpretation that separation impacts positively on outcome, our data can, however, also be marshalled to suggest that a negative effect ensued from the residency permit remaining a possibility or from even being granted. If this, admittedly somewhat counterintuitive, conjecture is correct, it could, due to groups overlapping, explain the poor outcome in the non-separated group. In line with this interpretation, the stress of seeking legally awarded compensation for an injury or illness has been proposed a unique contributor to symptom exaggeration, and de novo functional symptom generation [33]. It has been noted that such aggravation, or symptoms, sometimes fail to be reversed even after a favourable settlement has been reached, especially where legal procedures were longstanding thus involving multiple challenges of symptoms, and where improvement could threaten to reduce compensation. Hence, in parallel, it may be proposed that focusing on the residency permit, and in particular invoking it as “essential” to recovery, results in symptom hardening, and or symptom generation which come to persist as long as the chance of a successful application for residency remains, or even after it has been granted if improvement is perceived to come at the risk of a reversed decision. This effect may, if factual, be mediated by the caregivers and thus an expression of a harmful family dynamic. If so, lack of separation as well as focus on residency contribute to symptoms arising and persisting. Conceivably, these effects are subsumed under a notion of remodelled priors serving to alter behviour and ideation in response to contextual cues in line with a conception of culture-bound illness [16].

The main weaknesses of this study are the retrospective and naturalistic design, and recruitment. We do not know to what extent the participants are representative of RS patients. Participants may have been selected as a consequence of not responding to the current practice of family support and awaiting residency permit, but also for being suitable for Solsidan, which both could introduce a bias towards better response. Compulsory care in five cases indicates a possible difference from other samples. The diagnostic procedures, differential diagnostics and record keeping have been naturalistic and thus of a variable quality. This is partly overcome by data being elicited from records in a structured way. Further, the numbers are small and the observed time period was long, which adds to the risk of hidden factors driving outcome. Moreover, key analyses were constructed post hoc as a result of unexpected findings introducing added uncertainty. A randomised intervention study of sufficient size would supply stronger evidence. Also, due to difficulties identifiying former patients once discharged, we were unable to obtain follow-up data and hence cannot report long-term development and possible relapses.

In recognition of these weaknesses, our study may be perceived as a pilot and results should be interpreted with caution. Well aware of the limitations, we argue that great and long-term suffering, inefficient treatment and a scarcity of opportunities to acquire *any* data on resignation syndrome patients trump objections and motivate the study, in spite of the limitations presented. Its realisation was compelled by urgency.

That a residency permit is necessary for recovery, as stated in the treatment guideline issued by the Swedish National Board of Health and Welfare [9], needs to be challenged in recognition of the poor outcome in the group granted residency. Moreover, lack of treatment progress should no longer be excused by denied or uncertain residency status.

Contextual factors have been deemed sufficiently powerful to elicit and maintain the symptoms characterizing RS, and indeed to account for the phenomenon as such [16, 34, 35]. Thus, regardless of whether it is best conceived of as a culture-bound conversion syndrome, where expectations alter behaviour and ideation [16], or malingering by proxy–models that both can accommodate the regional distribution (endemic only to Sweden) and selective affliction (striking particular immigrant groups)–neutralizing harmful contextual elements appears fundamental in pre-empting the RS endemic. The possible harm resulting from a recommendation anchoring recovery to the outcome of a legal procedure–creating incentives for parents to intentionally or unintentionally instigate symptoms in their children, and children to intentionally or unintentionally take on the responsibility to improve the family’s chances of residency–needs to be recognised and counteracted. The pressing question is to what extent RS is a consequence of the recommended treatment method–the residency permit.

The national treatment guideline advocates a family-psychiatric approach [9] whereas our data suggest that the family in fact might stand in the way of recovery. This practice needs to be challenged in the light of our findings.

In appreciation of the heterogenous phenomena seemingly underlying the diagnosis of RS in this study–in particular three cases of suspected malingering by proxy–it is good news that the studied intervention provided a cure for RS regardless of its genesis*.* We don’t know to what extent the heterogeneity generalises to the whole group of RS patients. However, the intervention appears to benefit patients and families afflicted by RS in general and clinicians should consider it in all cases.

## Concluding remarks

The data suggests that residential therapy in separation from parents while abstaining from invoking residency is superior to the conventional method. Therefore, the family-psychiatric approach needs to be challenged. Moreover, treatment and pre-emptive strategies need to accommodate the harm possibly resulting from relying on the outcome of a legal process in treatment. Vigilance towards malingering by proxy is necessary. A lack of treatment response cannot be excused with reference to residency pending or being denied. We have now presented support for an active intervention and there is currently no evidence for a continued passive approach in RS.

Our data, in spite of methodological weakness, raise doubt about the present recommendations and suggests that these might further harm children suffering from RS.

## Supplementary Information

Below is the link to the electronic supplementary material.

Supplementary file1 (PDF 190 KB)

## Data Availability

There is no ethical approval for data sharing.

## Code availability

N/A.
