# Supplementary material for: Separation and not residency permit restores function in resignation syndrome: a retrospective cohort study
Source: Eur Child Adolesc Psychiatry. 2021 Jul 5;32(1):75–86. doi: 10.1007/s00787-021-01833-3 (PMC9908637; doi:10.1007/s00787-021-01833-3)
Supplement: Supplementary file 1 — Supplementary file1 (PDF 190 KB) [file 787_2021_1833_MOESM1_ESM.pdf]

Separation and not Residency Permit Restores Function in Resignation Syndrome: A Retrospective Cohort Study. Supplementary Material. Criteria for Resignation Syndrome (Uppgivenhetssyndrom).

Authors and affiliations

Karl Sallin*(1, 2, 3) MD, Kathinka Evers (1) PhD, Håkan Jarbin (4) MD, Lars Joelsson (5) MD, Predrag Petrovic (2) MD

1. Centre for Research Ethics & Bioethics (CRB), Uppsala University, P.O. Box 564, SE-751 22 Uppsala, Sweden
2. K8, Department of Clinical Neuroscience, Karolinska Institutet, SE-171 77 Stockholm, Sweden
3. Q2:07, Department of Paediatric Neurology, Astrid Lindgren Children's Hospital, Karolinska University Hospital, SE-171 76 Stockholm, Sweden
4. Lund University, Faculty of Medicine, Department of Clinical Sciences Lund, Child and Adolescent Psychiatry, Box 117, SE-221 00 Lund, Sweden
5. Child and Adolescent Psychiatric clinic, Uddevalla Hospital, Fjällvägen 9, SE-451 53 Uddevalla, Sweden

* Corresponding author at Centre for Research Ethics & Bioethics (CRB), Uppsala University, P.O. Box 564, SE-751 22 Uppsala, Sweden, or karl.sallin@crb.uu.se

Karl Sallin ORCID id: 0000-0003-0830-5645

Supplementary material. Criteria for Resignation Syndrome (Uppgivenhetssyndrom).

During the review process a clear presentation of the diagnosis was requested together with an examination of differential diagnoses elucidating differentiating and overlapping signs and symptoms. This request accords with a discussion recently offered with regards to Pervasive refusal syndrome [1]. We welcome this proposal. However, for resignation syndrome, this task is unfortunately not easily performed in particular since there are no official diagnostic criteria. Consequently, in order to perform the requested analysis, we would first have to endorse a proposal of diagnostic criteria. This would require the criteria to be duly validated, an endeavor well beyond the scope and format or this paper. Furthermore, the analysis would distract from the presentation of our results. However, in order honor the suggestion made in review, we contribute by providing a short background together with translations of three suggestions of criteria for resignation syndrome, previously, to our knowledge, only available in Swedish.

BACKGROUND

When the Swedish National Board of Health and Welfare issued the diagnostic code for resignation syndrome in 2014 it was specifically left to the professional bodies to establish criteria for the diagnosis. As of today, no official criteria exist. Consequently, the criteria

presented in the following are best conceived of as preliminary in anticipation of a due process addressing this issue.

There are three sets of criteria put forward for resignation syndrome. One set is presented in a Swedish Government Official Report [2]. The other two are referenced and presented by the Swedish National Board of Health and Welfare in a national treatment guideline [3] although originally presented elsewhere. All three sets are now presented in translation from Swedish into English and thus for the first time, we believe, made available to an international readership. Each is presented together with a short comment.

The first is the mobile asylum team in Stockholm county (MAsT) scale developed in 2005 and based on a “Simple evaluation of function in children with resignation syndrome” [4]. The second is the set from 2006 published in a Swedish Government Official Report [2]. The third is the “Bodegård’s criteria” [5] from 2008. Alongside these, the criteria for pervasive refusal syndrome [6] have also been used, although controversially (see [7, 8] for discussion).

A critical and comprehensive examination of the diagnosis is, together with a presentation of the differential diagnoses, also available in [7].

According to the Swedish National Board of Health and Welfare, the differential diagnoses for resignation syndrome include starvation, dehydration, brain tumor, meningitis, encephalitis, seizures, suicidal intoxication, depression, anxiety syndromes, anorexia nervosa and other eating disorders, selective mutism, catatonia, stupor, PTSD, dissociative disorder, somatoform disorder, conversion disorder, phobia (school), chronic fatigue syndrome, neurological disorders and Münchhausen-syndrome by proxy [3]. The Swedish Agency for Health Technology Assessment and Assessment of Social Services recognize several of the preceding however add to these, simulation [9].

THE MAsT EVALUATION SCALE

Here follow the MAsT evaluation scale translated literally from [3]. Also the Swedish version contains a repetition in the last section. As can be noted, these criteria were not intended to serve a diagnostic purpose.

The MAsT evaluation scale

The evaluation of function and the grading of children with resignation syndrome in three categories should not be perceived as a diagnostic instrument, but instead as a foundational prerequisite serving to illuminate the psychiatric and medical needs of these children, in other words a target group definition. The child and adolescent psychiatric diagnosis may still amount to for instance depression, PTSD or PRS. Another particularly important aspect of the evaluation of function has been that it has enabled a common language use between the different clinical disciplines.

Grading

Grade 1. Depression
Children exhibiting obvious signs of depression are at risk of entering a devitalised state. These children are passive, display little interest in other people, and their motor function is slow or characterised by uneasiness. The apatite is poor however, the child ingests sufficiently of food and fluids. The child performs also to some extent her daily routine, however with indifference and disinterest.

Grade 2. Towards resignation/apathy
A child approaching a devitalised state makes poor contact, can nod in response or respond in one syllable, and possibly reacts to some events. The mobility is reduced, as the child has to be encouraged to move or be helped or supported when moving within or outside of the residence. Also, the apatite is reduced; the parents have to encourage the child to eat as it exhibits little interest in food or is void of hunger. The daily routine is maintained by the parents or through their appeal.

Grade 3. Resignation/apathy
The condition implies that the child is inapproachable, closes her eyes or looks at the floor, and displays little or no interest in the outside world. Even the mobility is markedly reduced, the child lies down mostly and needs help to move. Ingestion requires a nasogastric tube or feeding by the parents. Furthermore, the child finds it difficult or close to impossible to perform a daily routine including personal hygiene and dressing; she may wet or soil herself or she is mostly unaware of such bodily signals and can’t dress without the assistance of someone else.

Instrument for evaluating loss of function in asylum seeking children and adolescents with resignation syndrome

Here follow the criteria from a Swedish Government Official Report literally translated from [2]. They were introduced to rate the level of functional loss and not for diagnostic purposes.

Instrument for evaluating loss of function in asylum seeking children and adolescents with resignation syndrome

Symptom	Grade I	Grade II
a) Capacity to communicate	Non-verbal response<br>i.e. nods or lowers gaze	No response<br>i.e. no words or eyes closed
b) Capacity to keep a daily routine	Performs task when urged<br>dresses, brushes teeth	No capacity to self-care<br>(needs to be dressed; enuresis; encopresis
c) Capacity to move	May be urged to walk assisted	Supine, can't support own weight standing; falls; no grimacing
d) Fundamental survival function	Fed, chews and swallows unassisted	Tub-fed
e) Aware of surroundings	E.g. hears or reacts to some events, may sometimes raise gaze	Completely shielded from surroundings

"BODEGÅRD'S CRITERIA"

It should be noted that the original Swedish formulations sometimes lack in precision. For instance, under 10 it is unclear how to interpret "hypnagogic jerks (Startle reactions)" as these are normally considered to be different phenomena. Also, under 11, "Stress hormone pathology" is undefined. Further, 2 b seems to hold a tautology and amount to little more than 2 a. This classification was not accepted by the National Board of Health and Welfare as it was considered too complicated [5]. Below, the criteria translated literally from [5] are presented.

Bodegård's criteria

Clinical evaluation criteria

Eleven symptoms grouped in four domains are presented here in order to enable classification of morbidity. Each symptom is graded from subtle (normal font) to severe (bold font). The criteria can be summed to one out of four diagnostic proposals.

I Contact

1. Abnormal contact – **No contact**
2. Unresponsive when addressed – Unresponsive to sudden noise – **Unresponsive to physical stimuli or pain**
3. **When approached the child reacts with severe active refusal, with aggression and or anxiety and or appears continuously in an anxious state more so than in hypotonic apathy**

II Motor function

4. Moves very little – Foetal position – **Perfectly still without muscle tone with eyes shut or open nevertheless seemingly "unseeing"**
5. The child is completely quiet
6. **Intermittent attacks of anxiety, whining, "howling" and or hyperventilation – spontaneous flashbacks/nightmares and hyperactivity when provoked**

III Nutrition and ADL

7. No spontaneous eating but can be fed – **Requires tube feeding to sustain**
8. Eliminates with assistance – **Urine and or faecal incontinence**

IV Miscellaneous

9. **Secondary somatic symptoms such as elevated temperature, tachycardia, transpiration, hyperventilation**
10. **In remission significant motor deficits despite obvious volition and intention – Hypnagogic jerks (Startle reactions) – Significant fatigue**
11. **Complications related to long-term care from muscles, skin and or kidneys, alopecia. Metabolic disturbances and stress hormone pathology**

1. Resignation syndrome mild

Symptoms from each of I to III (normal font) lasting more than four weeks regardless of diurnal or other variation. Short episodes of resignation syndrome symptomatology should not be considered diagnostic however constitute clinically important events.

2 a. Resignation syndrome severe, devitalisation (Depressive devitalisation)

One or more symptoms (bold font) from I to III with a duration of more than four weeks, conspicuously stable and eliciting a paediatric assessment resulting in intravenous hydration and nutrition, or tube feeding. The condition has been preceded by severe psychiatric symptoms and or have developed from resignation syndrome mild. Presentation can also be instant. It is life threatening unless nutrition is secured.

2 b. Resignation syndrome Devitalisation with refusal

Symptoms and course as in Depressive devitalisation with a clear element of 2 a.

2 c. Resignation syndrome Devitalisation with hyperreactivity and anxiety

Symptoms and course as in Depressive devitalisation with symptom 6 dominating.

REFERENCES

1. Schieveld JNM, Sallin K (2021) Pervasive refusal syndrome revisited: a conative disorder. Eur Child Adolesc Psychiatry 30:1–3. https://doi.org/10.1007/s00787-020-01685-3
2. Hessle M, Ahmadi N (2006) Asylsökande barn med uppgivenhetssymtom - trauma, kultur, asylprocess. SOU 2006:49. Statens Offentliga Utredningar, Stockholm
3. Socialstyrelsen (2013) Barn med Uppgivenhetssyndrom. En vägledning för personal inom socialtjänst och hälso- och sjukvård. Socialstyrelsen, Stockholm
4. Wiberg L (2005) Från uppgivenhet till skolstart Stockholms läns landstings vårdkedja för barn med uppgivenhetssymtom Rapport 1
5. Godani G, Bodegård G, Rydelius P-A (2008) Bördan de kom med. Karolinska Institutet. Save the Children Sweden, Stockholm
6. Jaspers T, Hanssen GMJ, Van Der Valk J a., et al (2009) Pervasive refusal syndrome as part of the refusal-withdrawal-regression spectrum: Critical review of the literature illustrated by a case report. Eur Child Adolesc Psychiatry 18:645–651. https://doi.org/10.1007/s00787-009-0027-6
7. Sallin K, Lagercrantz H, Evers K, et al (2016) Resignation Syndrome: Catatonia? Culture-Bound? Front Behav Neurosci 10:7. https://doi.org/10.3389/fnbeh.2016.00007
8. Von Folsach LL, Montgomery E (2006) Pervasive refusal syndrome among asylum-seeking children. Clin Child Psychol Psychiatry 11:457–473. https://doi.org/10.1177/1359104506064988
9. SBU (2020) Diagnostik och behandling av uppgivenhetssyndrom hos barn. SBU 2019/664.
